# Oral Hygiene Status, Tooth Loss, and Periodontal Disease Severity in Mexican Adults: A Cross‐Sectional Study

**DOI:** 10.1155/ijod/6842750

**Published:** 2026-01-23

**Authors:** García Pérez Alvaro, Mora Navarrete Karen Angelina, Rodríguez Chávez Jacqueline Adelina, Rodríguez González Kathia Guadalupe, Jiménez Núñez José Cuauhtémoc

**Affiliations:** ^1^ Laboratory of Public Health Research, Faculty of Higher Studies (FES), Iztacala, National Autonomous University of Mexico (UNAM), Tlalnepantla, State of Mexico, Mexico, unam.mx; ^2^ Department of Comprehensive Dental Clinics, University Center for Health Sciences, University of Guadalajara, Guadalajara, Jalisco, Mexico, udg.mx; ^3^ Veracruz Health Services, Veracruz, Mexico

**Keywords:** educational level, oral hygiene, periodontal disease, periodontitis, tooth loss

## Abstract

**Objective:**

Periodontal disease is a chronic condition, and its etiology is mainly related to biofilm and the destruction of the attachment apparatus resulting from poor application of oral hygiene measures. The aim was to study the relationship between oral hygiene and the severity of periodontal disease, in addition to evaluating the relationship between periodontitis and tooth loss in Mexican adults aged 20–49 years.

**Methods:**

Information from 10,897 Mexican adults between 2019 and 2022 was included by the Epidemiological Monitoring System for Oral Pathologies. The severity of periodontal disease was evaluated via the Community Periodontal Index (CPI) and classified into four groups: CPI = 0 (healthy); CPI = 1 (bleeding on probing); CPI = 2 (calculus); and CPI = 3/4 (periodontitis).

**Results:**

Of the total population, 20.4% presented bleeding, 30.3% presented dental calculus, 3.5% presented periodontitis, and 37.2% presented at least one missing tooth. Poor oral hygiene [OR = 4.01 (95% CI 3.24–4.96); *p*  < 0.001] and a low level of education [OR = 1.39 (95%CI 1.11–1.73); *p* = 0.003] were significantly associated with periodontitis. Adults with periodontitis are more likely to present tooth loss [OR = 2.51 (95% CI 2.01–3.15); *p*  < 0.001], with the probability of presenting the disease increasing in line with age.

**Conclusion:**

Poor oral hygiene and tooth loss are related to periodontitis in Mexican adults. It is essential to design strategies that strengthen motivation and promote oral health, with the goal of reducing the prevalence of periodontal disease and improving the maintenance of the dental support system.

## 1. Introduction

On a global level, periodontal disease is one of the main oral diseases affecting the population, with epidemiological studies reporting a higher prevalence and severity of periodontal disease, which affects up to 90% of the population when including gingivitis and 20% to 50% of other populations, depending on the diagnostic criteria used for their evaluation [1, 2]. The Global Burden of Disease Study 2021 reported that more than a billion people presented severe periodontitis, with a standardized global prevalence of 12.5% [3]. In addition, between 1990 and 2019, a negative impact on disability‐adjusted life years (DALYs) of periodontal disease has been observed [4].

Periodontal disease is a chronic inflammatory condition produced by the accumulation of biofilm and dental calculus and impacting the supporting tissues of teeth, such as the gums, periodontal ligament and attachment, root cement, and alveolar bone. If not treated, the disease evolves into periodontitis [5]. The signs comprise bleeding, inflammation, suppuration, and, occasionally, pain. Furthermore, it is one of the main causes of tooth loss, compromising speech, mastication, and deglutition, presenting negative consequences for self‐esteem and quality of life from an emotional, psychological, functional, and social perspective [6–8].

Among the risk factors for the presence of periodontal disease are metabolic factors (for example, diabetes and obesity), non‐modifiable factors (such as advanced age, genetic polymorphisms, and being male), and lifestyle factors (such as smoking, alcohol consumption, and poor diet) [9–11]. Further to the risk factors mentioned, a series of external factors, such as social inequalities, lack of access to healthcare services, a low level of education, a low socioeconomic level, and living in a rural area, affect oral health [12]. Then as well, poor oral hygiene directly contributes to periodontitis. It has been demonstrated that poor oral hygiene is a risk factor for the development of periodontal disease, given that the accumulation of plaque, which is the principal etiological agent, initiates and promotes gingival inflammation, thus impacting periodontal attachment [13]. As inflammation advances, the subgingival microbiome presents a higher level of Gram‐negative anaerobe production and the development of immune‐inflammatory responses that drive the destruction of periodontal tissue, which leads to the development of a periodontal pocket that, ultimately, advances to tooth loss [14, 15].

Mexico is a densely populated country with a total population of 126 million inhabitants, of whom 21% reside in rural areas, with a life expectancy of 68.9 years and total health expenditure reaching 52% of household income [16]. While there is a lack of complete information on the state of oral health in the Mexican population, some cross‐sectional studies have reported data on the prevalence of periodontal problems, which ranges from 20% to 89.0% [17, 18]. Likewise, dental plaque accumulation in the gingival margin is the primary risk factor for the presence of gingivitis and development of periodontitis. Periodontal disease can be prevented by proper oral hygiene and the removal of dental plaque, both by the dentist and the patient. In addition, it can be successfully treated with both ease and a minor financial impact on the patient when diagnosed early. This highlights the importance of identifying the required planning, diagnostic, and therapeutic strategies to promote both periodontal health and general well‐being in Mexican adults. Therefore, the objective of the present study was to explore the relationship between oral hygiene and the severity of periodontal disease, in addition to evaluating the relationship between periodontitis and tooth loss in Mexican adults aged 20–49 years.

## 2. Material and Methods

### 2.1. Design and Population Study

The present retrospective cross‐sectional study (using non‐probability convenience sampling methods) followed the STROBE criteria. Following the principles of the Declaration of Helsinki and authorized by the Ethical Review Board (CE/FESI/032023/1587) of the Iztacala Faculty of Higher Studies at the National Autonomous University of Mexico.

### 2.2. Data Collection

The periodontal status of the 20–49‐year‐old adults participating in the present research was evaluated using data obtained from the SIVEPAB, or Epidemiological Monitoring System for Oral Pathologies, which pertains to the General Directorate for Epidemiology at the Mexican Ministry for Health 2019–2022 [19]. The *inclusion criteria* for the adults were ≥20 years of age, women/men, and not presenting missing data. *Exclusion criteria* were presenting erupted third molars [19].

### 2.3. Independiente Variable

Oral hygiene was recorded using the Oral Hygiene Index‐Simplified (OHI‐S). Oral hygiene was dichotomized into poor oral hygiene (OHI‐S ≥ 2) or good oral hygiene (OHI‐S < 2) [20].

### 2.4. Dependent Variable: Periodontal Status

The present study used the Community Periodontal Index (CPI), a tool produced by the World Health Organization (WHO) to evaluate the periodontal health of a community, although it also describes the prevalence and gravity of periodontal conditions and helps to identify populations in need of periodontal treatment [19]. The CPI evaluates periodontal probing depth using the WHO probe with the following codes: CPI = 0 (Healthy); CPI = 1 (Bleeding on probing); CPI = 2 (Calculus); CPI = 3 (Pocket of 4–5 mm); and CPI = 4 (Pocket ≥ 6 mm deep) [19]. All the adults were examined by dentists in a dental chair fitted with a light source and a periodontal probe. The most severe CPI score for the sixths generated for each participant was registered as the final CPI score [19].

### 2.5. Covariables

Table 1 shows the variables that were considered by the present study according to the CPI.

**Table 1:** Distribution of the sample according to Community Periodontal Index (CPI) score among Mexican adults aged ≥20 years (*n* = 10,897).

Variables	CPI = 0 *n* = 4986 *n* (%)	CPI = 1 *n* = 2227 *n* (%)	CPI = 2 *n* = 3299 *n* (%)	CPI = 3/4 *n* = 385 *n* (%)	*p* Value ^∗^
Sex					
Women	1432 (28.7)	608 (27.3)	910 (27.6)	121 (31.4)	0.245
Men	3554 (71.3)	1619 (72.7)	2389 (72.4)	264 (68.6)	
Oral hygiene (OHI‐S)					
Good oral hygiene	3914 (78.5)	1123 (50.4)	1518 (46.0)	178 (46.2)	< 0.001
Poor oral hygiene	1072 (21.5)	1104 (49.6)	1781 (54.0)	207 (53.8)	
Level of education					
> 9 years	2517 (50.5)	1022 (45.9)	1459 (44.2)	147 (38.2)	< 0.001
≤ 9 years	2469 (49.5)	1205 (54.1)	1840 (55.8)	238 (61.8)	
Tooth loss					
No tooth loss	3375 (67.7)	1365 (61.3)	1945 (59.0)	154 (40.0)	< 0.001
≥ 1 tooth	1611 (32.3)	862 (38.7)	1354 (41.0)	231 (60.0)	

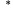
Chi‐square test.

### 2.6. Sample Size

It was obtained through the formula for a two‐proportion *Z*‐test. Supposing that 21% of the participants presented periodontitis, the study required a sample size of 172 participants per group with a power of 80% and a difference, in the proportions, of 0.14 between the two groups (bilateral *p* value of 0.05) [21].

### 2.7. Statistical Analysis

All data obtained were processed using the Stata 18 program (Stata Corp, College Station, TX, USA). Firstly, the characteristics of the adults were compared according to the severity of periodontal disease, with the associations between the groups then examined via the Xi‐squared test. Second, a multinomial regression analysis was conducted to determine the association between the independent variable and covariates and periodontal status [CPI = 0; CPI = 1; CPI = 2; and CPI = 3/4]. Possible interactions between oral hygiene and education were evaluated. Third, a binary logistic regression model was undertaken to examine the relationship between periodontitis and tooth loss, controlling for age and oral hygiene. The Odds Ratio (OR) was calculated with confidence intervals of 95%. Values of *p*  < 0.05 (two‐tailed) were considered statistically significant for all the analysis conducted. For the goodness‐of‐fit of the logistic model, the Hosmer–Lemeshow test was used.

## 3. Results

### 3.1. Study Population

10,897 Mexican adults aged 20–49 years participated; 59.5% were women with a mean age of 30.8 (±8.0) years. Significant differences were found when comparing mean ages by sex (men 32.2 vs. women 30.3; *p*  < 0.001), respectively. 61.7% of the participants presented poor oral hygiene, 52.8% had ≤ 9 years of formal education, and 37.2% presented at least one missing tooth, giving an average of 1.19 (±2.3).

**Figure 1:**
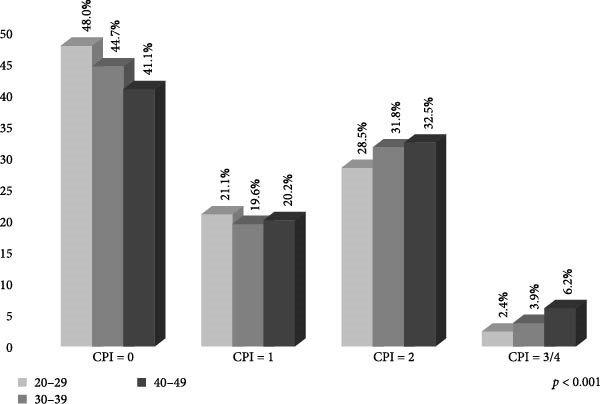
Percentage distribution of periodontal disease severity using the Community Periodontal Index (CPI) by age group in Mexican adults (*n* = 10,897).

**Figure 2:**
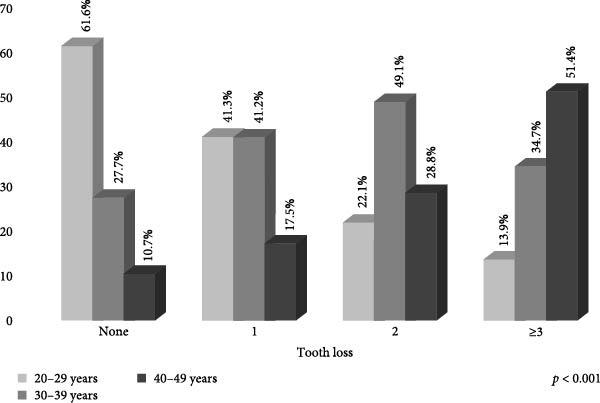
Percentage distribution of tooth loss by age group in Mexican adults (*n* = 10,897).

The severity of periodontal status was distributed as follows: 45.8% healthy (CPI = 0); 20.4% bleeding on probing (CPI = 1); 30.3% dental calculus (CPI = 2); and 3.5% periodontitis (CPI = 3/4). Table 1 presents information on adults according to the periodontal disease. The periodontitis was more prevalent in adults with poor hygiene (53.8%), a low level of education ( ≤ 9 years) (61.8%), ≥ 1 tooth (60.0%) and men (68.6%). Figure 1 shows the CPI categories by age group, wherein the 40–49‐year‐old group presented a higher percentage for dental calculus; moreover, it shows that the presence of periodontitis increased in line with age (*p*  < 0.001). Figure 2 shows that the 30–39 and 40–49‐year‐old groups had a higher percentage of tooth loss ( ≥ 3 teeth), and it is also observed that the presence of tooth loss increased in line with age (*p*  < 0.001).

Variables such as age, poor oral hygiene, and low education ( ≤ 9 years) were related to the presence of bleeding on probing, calculus, and periodontitis (*p*  < 0.001) (Table 2). The results of the logistic model to determine the variables associated with tooth loss are shown in Table 3. Significant associations were observed with the following variables: age, poor oral hygiene, and periodontitis with tooth loss (*p*  < 0.001). Moreover, the probabilities of tooth loss in adults increased in line with both age and the severity of periodontal disease (*p*  < 0.001).

**Table 2:** Multinomial logistic regression model for the association between oral hygiene status and severity of periodontal disease among Mexican adults (*n* = 10,897).

Variables		CPI = 0	CPI = 1	CPI = 2	CPI = 3/4
			Odds ratio (95%CI)	Odds ratio (95%CI)	Odds ratio (95%CI)
Sex	Men	Reference	Reference	Reference	Reference
	Women	*Reference*	1.04 (0.93–1.17) *p* = 0.455	1.04 (0.93–1.15) *p* = 0.443	0.91 (0.72–1.14) *p* = 0.446

Age	20–29 years	Reference	Reference	Reference	Reference
	30–39 years	Reference	0.96 (0.86–1.08) *p* = 0.559	1.15 (1.04–1.27) *p* = 0.006	1.69 (1.32–2.17) *p* < 0.001
	40–49 years	Reference	1.01 (0.87–1.17) *p* = 0.821	1.18 (1.04–1.35) *p* = 0.011	2.60 (1.98–3.41) *p* < 0.001

Oral hygiene (OHI‐S)	Good oral hygiene	Reference	Reference	Reference	Reference
	Poor oral hygiene	Reference	3.56 (3.20–3.96) *p* < 0.001	4.21 (3.82–4.64) *p* < 0.001	4.01 (3.24–4.96) *p* < 0.001

Level of education	> 9 years	Reference	Reference	Reference	Reference
	≤ 9 years	Reference	1.12 (1.01–1.24) *p* = 0.029	1.17 (1.06–1.28) *p* = 0.001	1.39 (1.11–1.73) *p* = 0.003

Abbriviations: CI, Confidence Interval; OR, Odds ratio.

**Table 3:** Adjusted odds ratios from the logistic regression model for tooth loss and severity of periodontal disease among Mexican adults (*n* = 10,897).

Variables		Crude OR (95% CI)	*p*	Adjust OR (95% CI)	*p*
Age	20–29 years	Reference		Reference	
	30–39 years	2.93 (2.68–3.21)	< 0.001	2.89 (2.64–3.17)	< 0.001
	40–49 years	4.90 (4.38–5.48)	< 0.001	4.72 (4.21–5.28)	< 0.001

Oral hygiene (OHI‐S)	Good oral hygiene	Reference		Reference	
	Poor oral hygiene	1.36 (1.25–1.47)	< 0.001	1.16 (1.06–1.27)	0.001

Severity of periodontal disease	CPI = 0	Reference		Reference	
	CPI = 1 (bleeding gingival)	1.32 (1.19–1.46)	< 0.001	1.27 (1.14–1.42)	< 0.001
	CPI = 2 (calculus)	1.45 (1.33–1.59)	< 0.001	1.33 (1.20–1.47)	< 0.001
	CPI = 3/4 (periodontitis)	3.14 (2.54–3.88)	< 0.001	2.51 (2.01–3.15)	< 0.001

*Note*: Log likelihood = −6625.5627, Hosmer‐Lemeshow test *p* = 0.0891

Abbriviations: CI, confidence interval; OR, odds ratio.

## 4. Discussion

The present cross‐sectional study reported that poor oral hygiene increased the odds of presenting bleeding on probing, dental calculus, and periodontitis, as evaluated via the application of the CPI. The accumulation of dental plaque and dental calculus has been considered a risk factor in the onset of gingivitis and its progression to periodontitis, mainly due to inadequate oral hygiene [22, 23]. While the literature reports that dental plaque comprising a community of bacteria and microorganisms is the initial cause of periodontal disease, it also describes how dental calculus plays a complementary function and acts solely in retaining plaque. Thus, it is eliminated solely in the interests of improving oral hygiene [24]. Notwithstanding, it has been reported that dental calculus plays an essential role in periodontal inflammation, progression, and destruction [24]. For example, a study conducted to explore the relationship between dental calculus and signs of the inflammation of the wall of the periodontal pocket found that subgingival calculus deposits covered in biofilm were related to more than 60% of the inflammation of the pocket wall [25]. Therefore, it is important that the elimination of dental calculus be undertaken via treatments able to effectively eliminate the supragingival and subgingival biofilm, such as certain toothbrushing techniques, interdental cleaning, and regular visits to the dentist. Said treatments should be combined with the use of antiseptic products and the provision of education programs to help modify the habits related to alcohol consumption and smoking to maintain periodontal health in adults.

The present study found that poor oral hygiene increased the odds of periodontitis by nearly four times (OR = 3.88; *p*  < 0.001). Poor oral hygiene is an established risk factor for periodontitis, with a meta‐analysis conducted on 50 studies having found that regular to poor oral hygiene increases the probability of periodontitis by between two and five times [OR = 2.04 (95% CI 1.65–2.53) and OR = 5.01 (95% CI: 3.40–7.39)], respectively [26]. It must be noted that oral hygiene plays an important role in periodontal status. Therefore, there is a great need for activities to be carried out to improve oral health, such as toothbrushing at least twice a day with fluoride toothpaste and visiting the dentist at least twice a year, in accordance with the recommendations of the World Dental Federation [27]. Time and resources should also be invested in fomenting interest in and communication about oral hygiene in the general population.

Periodontitis is a complex infectious disease that progresses over time due to the accumulation of dental plaque and the onset of bacterial dysbiosis that, if untreated, produces periodontal pockets, gingival recession, tissue destruction, and alveolar bone loss. As a consequence of said symptoms, the disease can cause tooth loss [28]. The presence of periodontitis increases the odds of tooth loss by 2.5 times in Mexican adults (OR = 2.51; *p*  < 0.001). The literature also reports similar results, wherein, for example, an adult population in Turkey was found to present a relationship between periodontitis and tooth loss (OR = 1.3; *p*  < 0.05) [29]. A retrospective study conducted on Japanese patients found that those diagnosed with periodontitis were at a higher risk of experiencing tooth loss [30]. It is of the utmost importance that clinicians promote oral health with their patients, given that this would contribute to improving periodontal maintenance and preventing periodontitis.

Mobile phone applications are one technological tool via which dentists can foster oral hygiene, a subject on which, moreover, a systematic review found that many applications are already focused. Some have been shown to be effective in highlighting the importance of toothbrushing and even educating the patient regarding the various existing toothbrushing techniques and the use of complementary products such as dental floss. The foregoing presents an area of opportunity for interventions with individuals of all ages, although mainly the young, since they are often more interested in and familiarized with this technology [31].

The present study identified a relationship between the severity of periodontal disease and a low level of education, as well as observing that the probability of presenting periodontitis increases in line with age, meaning that the increased rates of aging in the population have led to an increase in the prevalence of the condition [32]. Ordinarily, the severity of periodontal disease affects vulnerable populations of various socioeconomic levels and fewer years of schooling [19], with adults with a low level of education observed to present poorer health than other sections of the population, even in developed countries such as the United States [33]. In the same way, a meta‐analysis found that a low education was related to a higher possibility of periodontitis in adults aged ≥ 35 years [34]. The possible reasons for which Mexican adults present higher levels of oral health inequality that are related to a low level of education include the cost of dental visits, the costs of dental treatment, and the lack of access to dental health services [35, 36]. In general terms, a person’s educational level can have a determining effect on their health because it affects the development of the habits, skills, and resources that enable them to improve their health [37]. Accordingly, there is an essential for health education that helps to reduce the prevalence and incidence of oral health problems in the Mexican population. Furthermore, education promotes and maintains health‐conscious living, strengthens personal connections, and improves personal, family, and population well‐being [38].

The present study has some limitations. First, the cross‐sectional design for obtaining the information did not allow for a causal analysis. Secondly, as only data obtained from appointments at sentinel units was used by the present study, this generated a selection bias; however, the results obtained did provide a broad overview of the periodontal patients examined at these units. Thirdly, even though the information collected is not representative of the Mexican population and, thus, may minimize the estimated prevalence of periodontal disease, the narrow confidence intervals obtained indicate that the estimation is more precise. The findings primarily reflect adults attending sentinel units, rather than the entire Mexican adult population.

## 5. Conclusions

The present study found that poor oral hygiene is related to periodontitis in Mexican adults who assist the sentinel units; moreover, the presence of periodontitis increases the probability of tooth loss. A low level of education and advanced age increase the probability of presenting periodontitis. Therefore, it is of the utmost importance that, to reduce the prevalence of periodontal disease, improve oral health, and, thus, impact positively on quality of life, strategies are designed to both coincide with the needs of the population and strengthen the promotion of oral health.

## Ethics Statement

The research was carried out in accordance with the principles outlined in the Declaration of Helsinki and approved by the Ethics Committee of the Iztacala Faculty of Higher Studies at the National Autonomous University of Mexico (CE/FESI/032023/1587).

## Consent

The authors have nothing to report, since a retrospective study was conducted with available databases.

## Conflicts of Interest

The authors declare no conflicts of interest.

## Funding

The authors received no financial support for the research, authorship, and/or publication of this article.

## Data Availability

The data that support the findings of this study are available on request from the corresponding author, García Pérez Alvaro.
